# Iron Complexes with Antarctic Krill–Derived Peptides Show Superior Effectiveness to Their Original Protein–Iron Complexes in Mice with Iron Deficiency Anemia

**DOI:** 10.3390/nu15112510

**Published:** 2023-05-28

**Authors:** Shengjie Hu, Songyi Lin, Qi Feng, Xueqing He, Haowei Xu, Lei Chen, Na Sun

**Affiliations:** 1 School of Food Science and Technology, National Engineering Research Center of Seafood, Dalian Polytechnic University, Dalian 116034, China; hushengjie657@126.com (S.H.); linsongyi730@163.com (S.L.); fq010118@163.com (Q.F.); hexueqing1229@163.com (X.H.); xuhaowei98@163.com (H.X.); chenleisst@163.com (L.C.); 2 State Key Laboratory of Marine Food Processing and Safety Control, Dalian Polytechnic University, Dalian 116034, China

**Keywords:** IDA mice, Antarctic krill peptide–iron, iron-regulated genes, iron bioavailability, in vivo antioxidant capacity

## Abstract

Antarctic krill protein–iron complex and peptide–iron complex were acquired to investigate their iron bioavailability, expression of iron-regulated genes, and in vivo antioxidant capacity. Results indicated that the Antarctic krill peptide–iron complex significantly increased the hemoglobin (Hb), serum iron (SI), and iron contents in the liver and spleen in iron-deficiency anemia (IDA) mice (*p* < 0.05) compared with those of the Antarctic krill protein–iron complex. Despite the gene expressions of the divalent metal transporter 1(DMT1), the transferrin (Tf), and the transferrin receptor (TfR) being better regulated by both Antarctic krill peptide–iron complex and protein–iron complex, the relative iron bioavailability of the Antarctic krill peptide–iron complex group (152.53 ± 21.05%) was significantly higher than that of the protein–iron complex group (112.75 ± 9.60%) (*p* < 0.05). Moreover, Antarctic krill peptide–iron complex could enhance the antioxidant enzyme activities of superoxidase dismutase (SOD) and glutathione peroxidase (GSH-Px), reduce the malondialdehyde (MDA) level in IDA mice compared with the protein–iron complex, and reduce the cell damage caused by IDA. Therefore, these results indicated that Antarctic krill peptide–iron complex could be used as a highly efficient and multifunctional iron supplement.

## 1. Introduction

Many biological processes rely on iron, including oxygen transport and energy metabolism [1]. Iron deficiency can cause iron deficiency anemia (IDA), which is one of the most frequent nutritional deficiencies affecting millions of people globally [2]. Iron deficiency can decrease hemoglobin (Hb) and increase oxidative stress [3], which may contribute to the pathogenesis of IDA [4].

Ferric iron is generally accepted as the most prevalent form of non-heme iron. Cytochrome b on the apical surface of intestinal epithelial cells in the lumen of the gut reduces ferric iron to the ferrous form that is further transported to intestinal epithelial cells via DMT1 [5,6]. Iron is transported throughout the body via transferrin (Tf), which transfers iron to sites of absorption [7], where the cells take up transferrin-bound ferric iron via the transferrin receptor (TfR1) [8]. The bioavailability of dietary iron is very low, ranging from 5% to 18% [9], and intestinal iron absorption is influenced by the solubility [10]. This low bioavailability and issues with absorption can result in IDA. Therefore, several iron supplements have been developed to address this pressing public health issue [11,12]. As an iron supplement, FeSO_4_ has been approved in many countries under pharmaceutical and food additive regulations [13]. However, it can cause adverse reactions that affect the intestinal lumen and mucosa because of free iron, which leads to the production of free radicals [14,15]. Therefore, it is necessary to reduce interactions between free iron and cells in the gastrointestinal tract during digestion and absorption. In addition to reducing the toxicity of free iron, it is possible to increase its solubility and bioavailability. The absorption of intestinal iron is generally considered the important factor that controls iron levels [16]. Due to the fact that the excretion process of iron is not regulated by the organism and that iron is lost daily by the body, the body maintains homeostasis of iron through upregulation or downregulation of iron absorption mechanisms [17]. Hence, enhanced intestinal iron absorption is crucial for improving iron levels in blood and peripheral tissues during IDA.

Recently, iron supplements containing iron-chelating proteins and peptides have been developed. For example, lentil-derived protein–iron complex significantly reduced the expression levels of DMT1 and TfR genes in Caco-2 cells with IDA [18]. Ovalbumin promotes the recovery of hematological indices and liver iron content to normal levels in IDA rats [19]. Peptides from duck egg white also significantly enhance hematological indices in IDA rats [20]. In addition, iron uptake and ferritin levels in Caco-2 cells can be enhanced using barley peptides [21]. Our previous study showed that Antarctic krill peptides obtained by enzymolysis of Antarctic krill powder with trypsin were rich in aspartic and glutamic acids, which enhanced iron transport in Caco-2 cells after simulated gastrointestinal digestion [22]. Antarctic krill proteins are rich in essential amino acids and are more biological advantageous than other animal proteins [23]. Wang et al. found that after 3 h of trypsin digestion, the binding capacity of the Antarctic krill peptide to iron was nearly 80% [22], which was higher than that of the casein peptide (by nearly 50%) [24]. However, the effect of Antarctic krill peptides on iron absorption in vivo has not yet been reported. In addition, whether there is a difference in in vivo iron absorption from Antarctic krill peptides compared to Antarctic krill proteins needs to be investigated in greater depth.

For the purpose of experimenting which is more effective for the recovery of IDA between Antarctic krill protein–iron and peptide–iron complexes, this study prepared Antarctic krill proteins and peptides from Antarctic krill powder and evaluated the effects of Antarctic krill proteins and peptides on iron bioavailability, expression of iron-regulated genes, and in vivo antioxidant capacity.

## 2. Materials and Methods

### 2.1. Extraction of Antarctic Krill Proteins and Preparation of Antarctic Krill Peptides

Antarctic krill proteins were extracted by referring to Gao et al. [25]. Antarctic krill meat was added to distilled water with constant stirring and mixing. The mixture was adjusted to pH 11.5 and continually stirred at 4 °C for 2 h. Subsequently, the supernatant was obtained by centrifugation for 10 min at 3200× *g*. Thereafter, the supernatant was extracted for 2 h at 4 °C and pH 4.6 to collect the precipitate. The precipitate was freeze dried and labeled as Antarctic Krill protein. 

In addition, Antarctic krill peptides were prepared according to the method by Gao et al. [26]. First, Antarctic krill proteins were dissolved in double distilled water, followed by addition of alcalase (50 °C, pH 8.5) at a dose of 5000 U/g protein to hydrolyze the proteins for 3 h. In a water bath, the enzyme was inactivated by heating at 100 °C for 10 min. Subsequently, the supernatant was obtained by centrifugation at 10,000× *g* for 20 min at 4 °C and stored at −20 °C for later analysis.

### 2.2. Preparation of Iron Complexes with Antarctic Krill Proteins or Peptides

According to our previous method [26], freeze-dried Antarctic krill proteins or peptides were dispersed in double-distilled water at a concentration of 30 mg/mL. Subsequently, FeSO_4_ was added to a final concentration of 50 mM. The mixed system was shaken and reacted for 30 min at 25 °C and pH 6.0.

### 2.3. Animal Care and Treatment

Fifty 3-week old female C57/B6 mice weighing 13 ± 5 g were acquired from Liaoning Changsheng Biotechnology Co., Ltd. (Benxi, Liaoning, China). A specific-pathogen-free (SPF) environment (22 ± 2 °C, 60% ± 5% humidity, and 12 h of dark/light cycle) was maintained for the mice. The experimental mice were randomly divided into five groups consisting of ten mice in each group. Ten mice were fed with a standard diet (45 mg Fe/kg diet, Trophic Animal Feed High-tech Co., Ltd., Nantong, China) throughout the experiment as the control group. In the other experiments, the mice were continuously fed with a diet (12 mg Fe/kg diet, Trophic Animal Feed High-tech Co., Ltd., Nantong, China) for 8 weeks to obtain IDA mice models. To conduct routine blood tests, blood was collected using orbital venipuncture after 8 weeks The hemoglobin (Hb) value of less than 100 g/L was taken as indicative of an IDA. 

After the establishment of the IDA mice model, the four groups of mice were continuously fed with a low-iron diet for 3 weeks. the mice in the control and model groups were administrated deionized water every day. The positive control group was administered FeSO_4_ (2 mg Fe/kg BW), and the other two groups given Antarctic krill protein–iron complex and peptide–iron complex respectively (2 mg Fe/kg BW). The administration was intragastric to mice at the same time points, once a day for 3 weeks. Iron supplements used for gavage were dissolved in water, and prepared and used in a very short time on the same day to prevent oxidation. During a 3-week period of intragastric administration, food intake and body weight were measured weekly. In addition, mice were euthanized by isoflurane overdose followed by cervical dislocation, and all mice were culled at the same time of day after the last gavage of 18h. Serum, stomach, duodenum, liver, and spleen were collected. Animal ethics approval was granted by the Animal Ethics Committee of Dalian Polytechnic University (protocol number: DLPU2022024).

### 2.4. Hematological Test

The hemoglobin (Hb), serum iron (SI) concentration, and total iron binding capacity (TIBC) levels were measured using an Hb test solution, SI assay kit, and TIBC assay kit, respectively (Nanjing Jiancheng Bioengineering Inst., Nanjing, China).

### 2.5. Relative Biological Value and Hb Regeneration Efficiency 

It has been reported that in both anemic animals and animals recovering from IDA, that the blood volume of growing animals is about 6.7% of the body weight and Hb contains 0.335% (*w*/*w*) iron [27]. The related formula is shown as follows:

Hb iron pool (mg) = BW (kg) × 0.067 × Hb concentration (g/L) × 3.35

Hb regeneration efficiency (%) = [final Hb iron pool (mg) − initial Hb iron pool (mg)]

/total consumed iron (mg) × 100

Relative biological value (%) = Hb regeneration efficiency of each animal

/Hb regeneration efficiency of the FeSO_4_ group × 100

### 2.6. Analysis of Iron Content in Liver and Spleen

An amount of 0.1 g liver and spleen were digested with nitric acid/perchloric acid (20:1, *v*/*v*) at 300 °C for 30–40 min until the solution became clear. The content of iron was determined using inductively coupled plasma optical emission spectrometry (ICP-OES) (PerkinElmer, Waltham, MA, USA).

### 2.7. Real-Time (RT) PCR Analysis

A quantitative real-time polymerase chain reaction (RT-PCR) was used to measure liver DMT1, Tf, and TfR gene expression. GAPDH was used as the internal reference. DMT1, Tf, and TfR gene expression were measured by the ΔΔCt method in relation to *β*- actin levels. Table 1 displays the primer sequences. Quantitative RT-PCR was performed using three biological samples and three technical replications.

### 2.8. The Detection of the SOD, GSH-PX, and MDA Levels in the Gastric Tissue

An amount of 0.1 g of mice gastric tissue was taken, followed by homogenization with ice saline. Subsequently, the supernatant was obtained by centrifugation at 4000× *g* for 15 min at 4 °C. Kits (Nanjing Jiancheng Bioengineering Institute, Nanjing City, China) were used to determine the levels of superoxide dismutase (SOD), glutathione peroxidase (GSH-Px), and malondialdehyde (MDA) in the gastric tissue.

### 2.9. Histopathological Observation

First, the liver and spleen from each group of mice were soaked in 10% formalin. Tissue samples were then dehydrated with an ethanol gradient, cleared with xylene, and embedded in paraffin. Afterwards, tissue samples were stained with hematoxylin and eosin. Slices were observed with an optical microscope with a ×400 magnification.

### 2.10. Statistical Analysis

The experimental data were summarized as the mean ± SD, and inter-group significance was determine using one-way analysis of variance (ANOVA). Statistically significant differences between groups were determined using *p* < 0.05.

## 3. Results and Discussion

### 3.1. The Growth of IDA Mice during Iron Supplementation

**Figure 1:**
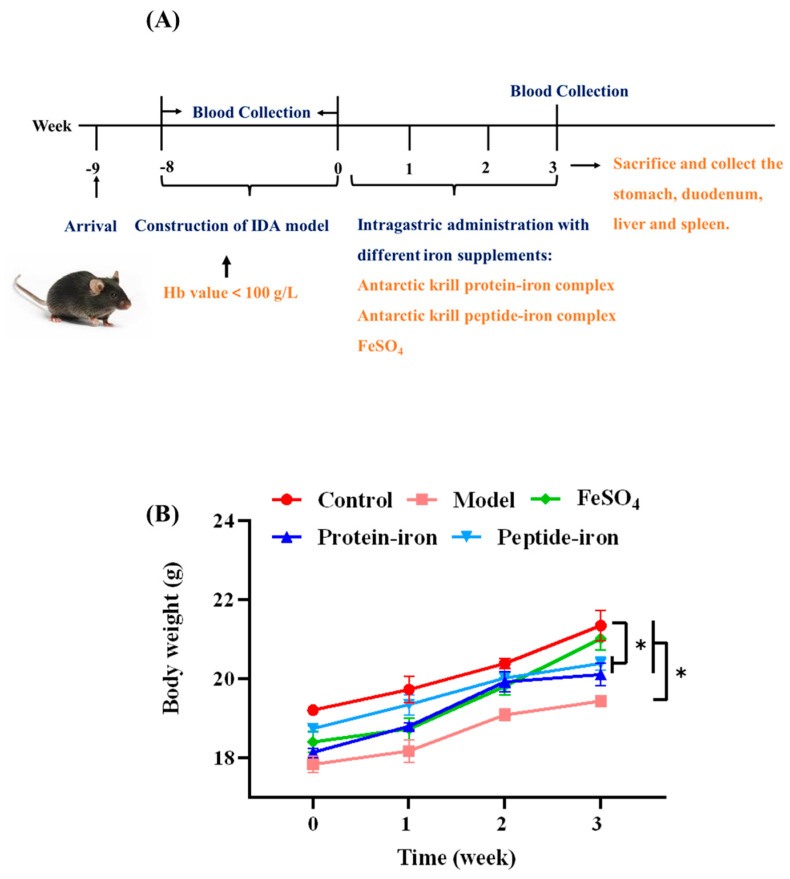
Mice experimental protocol and body weight changes in mice. (**A**) Mice experimental protocol: an iron−deficient diet was used for 8 weeks to establish an IDA mice model and mice were treated for 3 weeks with Antarctic krill peptide−iron complex, protein–iron complex or FeSO_4_; body weight and collected serum were regularly measured; at the end of the experiment, serum, stomach, duodenum, liver and spleen were collected; (**B**) the body weight changes after iron supplementation for different groups (control, model, Antarctic krill protein−iron complex, peptide–iron complex, and FeSO_4_ groups). SEM error bars are present. Significant differences are indicated by asterisks (*p* < 0.05).

In this experiment, an iron-deficient diet was used for 8 weeks to establish an IDA mice model. Mice were treated for 3 weeks with Antarctic krill peptide–iron complex, protein–iron complex or FeSO_4_. We regularly measured body weight and collected serum. At the end of the experiment, serum, stomach, duodenum, liver, and spleen were collected (Figure 1A). Mice in the IDA groups lost significant weight after 8 weeks of the iron-deficient diet (*p* < 0.05) compared with the weight of the control group (Figure 1B). There was no significant difference in body weight between the iron-deficient diet groups (*p* > 0.05). IDA mice had poor health during the development of the iron deficiency model, presenting with pale skin, rough hair, slow growth, and loss of appetite. Several studies reported that IDA can affect the growth of mice [12,28], which is consistent with our experimental results. Liu et al. [29] and Zielińska-Dawidziak et al. [30] reported similar signs of poor health in their animal models. The health of the animals in their study improved significantly with iron supplementation. At the end of the iron supplementation period (3 weeks), the treatment groups showed significant increases in body weight compared with the IDA group (*p* < 0.05), and there was no significant difference between the Antarctic krill protein–iron and peptide–iron complex groups (*p* > 0.05). 

### 3.2. Hematological Parameter Analysis

**Figure 2:**
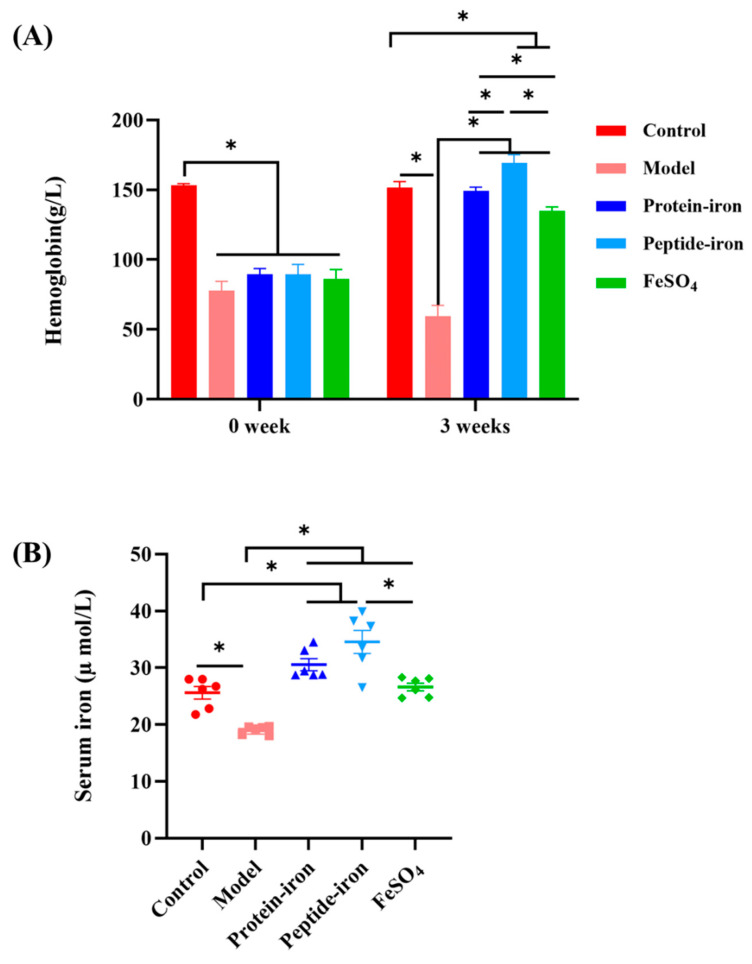

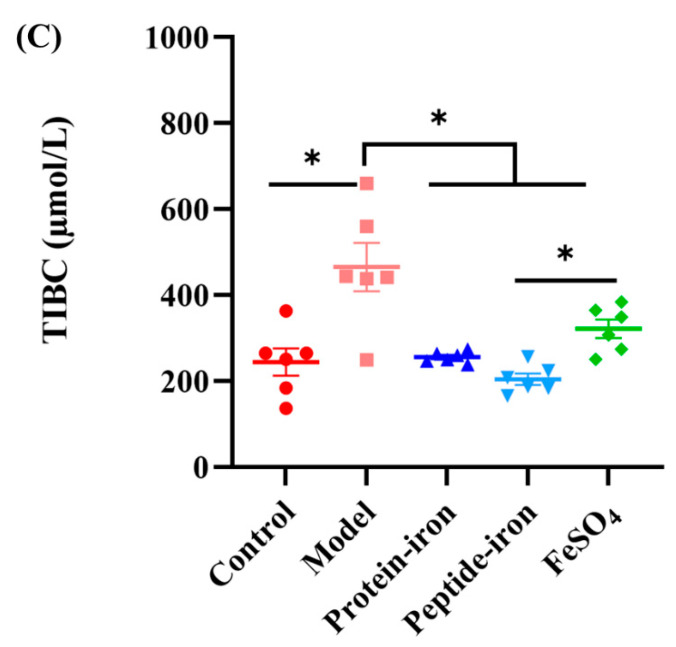
Hb, SI, and TIBC levels of the mice in different groups. (**A**) The changes in Hb concentration before and after iron supplementation for different groups (control, model, Antarctic krill protein–iron complex, peptide–iron complex, and FeSO_4_ groups); (**B**) the changes in SI concentration after iron supplementation for different groups (control, model, Antarctic krill protein–iron complex, peptide–iron complex, and FeSO_4_ groups); (**C**) the changes in TIBC levels after iron supplementation for different groups (control, model, Antarctic krill protein–iron complex, peptide–iron complex, and FeSO_4_ groups). Data are presented as mean ± SD. Significant differences are indicated by asterisks (*p* < 0.05).

In this study, we examined the hematological indicators associated with iron status, including hemoglobin (Hb), serum iron (SI), and total iron binding capacity (TIBC). Hb levels before and after iron supplementation are shown in Figure 2A. IDA can cause a decrease in Hb [31]. A significant difference in Hb levels was observed between the IDA and control groups (*p* < 0.05). This is because iron deficiency causes the functional iron level in the blood to decrease, which decreases Hb levels [32]. At the beginning of treatment, there were no significant differences in the Hb content between the iron-supplemented and IDA groups (*p* > 0.05). Following iron supplementation, the Hb levels of mice in the iron-supplemented groups were drastically enhanced and gradually approached those of the control group. The Hb concentrations of the Antarctic krill peptide–iron complex group (169.23 ± 5.87 g/L) and the protein–iron complex group (149.19 ± 2.78 g/L) were significantly higher than that of the FeSO_4_ group (135.13 ± 2.56 g/L; *p* < 0.05). Indeed, the Hb content can return to near normal levels with improved iron status [33].

The determination of the SI and TIBC is of great significance for the clinical diagnosis of IDA [34] SI indicates the amount of transferrin-bound iron in the circulation [35]. As shown in Figure 2B, IDA had a greater impact on SI concentration in mice, resulting in a lower SI concentration (19.05 ± 0.73 µmol/L). The Antarctic krill peptide–iron and protein–iron complexes significantly increased SI concentrations in mice with IDA (34.55 ± 4.96 µmol/L and 30.54 ± 2.57 µmol/L, respectively; *p* < 0.05). Mice in the FeSO_4_ group had the lowest SI concentration (26.60 ± 1.65 µmol/L) in the iron supplementation group. 

TIBC reflects the ability of blood to bind iron with transferrin and often increases during iron deficiency [36]. As seen in Figure 2C, IDA mice had significantly higher TIBC levels (465.92 ± 56.30 µmol/L; *p* < 0.05). After receiving iron supplementation, the TIBC levels in IDA mice were significantly reduced (*p* < 0.05) compared to those before supplementation, and all treatment groups had levels similar to that of the control group, with no significant differences compared to control (*p* > 0.05). A significant difference was observed in the TIBC level between the Antarctic krill peptide–iron complex group (204.95 ± 13.31 µmol/L) and the protein–iron complex (255.86 ± 5.35 µmol/L) and FeSO_4_ groups (322.04 ± 21.64 µmol/L; *p* < 0.05). 

The findings showed a dramatic improvement in Hb, SI, and TIBC levels after administration of the Antarctic krill peptide–iron and protein–iron complexes. Specifically, the Antarctic krill peptide–iron complex exhibited a better therapeutic effect. This may be related to the structure and amino acid composition of the Antarctic krill peptides. Wang et al. found that Asp, Glu, and His contents in Antarctic krill peptides were positively correlated with iron-binding activity after Antarctic krill powder was hydrolyzed by trypsin [22]. It has been shown that peptides rich in these amino acids promote iron absorption [37,38]. 

### 3.3. Iron Content in the Liver and Spleen of Mice

**Figure 3:**
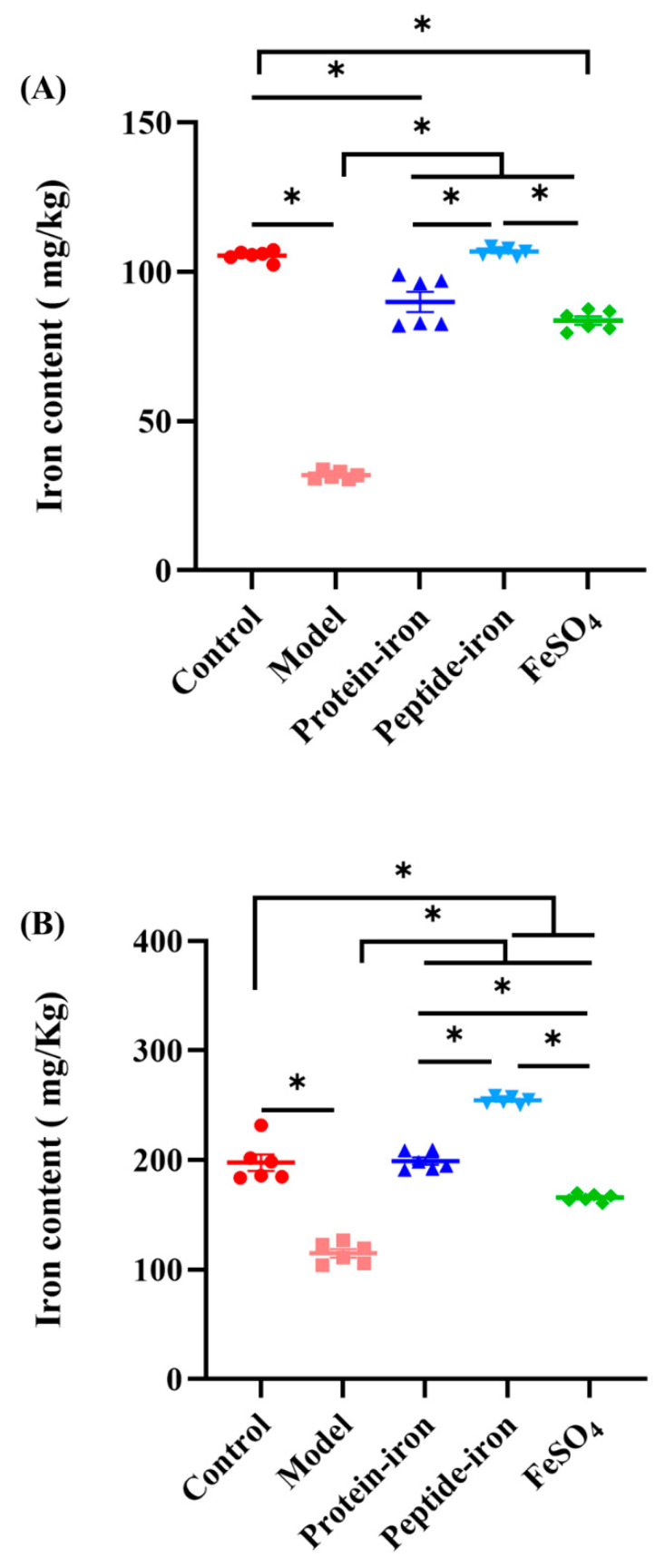
The changes of iron contents of mice in the liver and spleen after iron supplementation for different groups. (**A**) the changes of iron contents of mice in the liver after iron supplementation for different groups (control, model, Antarctic krill protein–iron complex, peptide–iron complex, and FeSO_4_ groups); (**B**) the changes of iron contents of mice in the spleen after iron supplementation for different groups (control, model, Antarctic krill protein–iron complex, peptide–iron complex, and FeSO_4_ groups). Data are presented as mean ± SD. Significant differences are indicated by asterisks (*p* < 0.05).

Figure 3 shows iron accumulation in the liver and spleen. The IDA mice had significantly lower iron content in the liver than the control group (31.93 ± 0.52 mg/kg vs. 105.49 ± 0.68 mg/kg). The iron content of the liver in the Antarctic krill peptide–iron complex group (106.766 ± 0.53 mg/kg) gradually approached the control group after iron supplementation. There was a significant increase in liver iron content in the Antarctic krill peptide–iron complex group compared to that in the Antarctic krill protein–iron complex (89.94 ± 3.37 mg/kg) and the FeSO_4_ groups (83.67 ± 1.34 mg/kg; *p* < 0.05). There was no significant difference between the protein–iron complex group and the FeSO_4_ group (*p* > 0.05). In addition, the iron content in the spleen showed a trend similar to that observed in the liver. The Antarctic krill peptide–iron complex group (254.54 ± 1.35 mg/kg) had a higher iron content in the spleen than the control (197.74 ± 7.46 mg/kg; *p* > 0.05), protein–iron complex (199.26 ± 3.30 mg/kg; *p* > 0.05), and the FeSO_4_ groups (165.80 ± 1.43 mg/kg; *p* < 0.05; Figure 3B). 

As the major iron storage tissues, the liver and spleen play crucial roles in iron homeostasis [39]. IDA mice use iron stored in the liver and spleen for transport to maintain serum iron balance [40]. Therefore, IDA mice generally have lower levels of liver iron than other groups. Similar results were reported by Zhang et al. [41]. Studies have confirmed that, to compensate for iron deficiency and Hb reduction, the body enters a hemolytic state, which leads to hypertrophy of the liver and spleen [31]. This phenomenon was observed in previous study [42]. In addition, the amino acid composition of the diet may affect iron accumulation in the liver and spleen. Ma et al. demonstrated that glutamate-rich peptides reduce transferrin production, which favors iron storage in the liver and spleen [43]. This may explain why peptide–iron complexes are more favorable for iron storage than protein–iron complexes.

### 3.4. Hemoglobin Regeneration Efficiency and Relative Biological Value 

**Figure 4:**
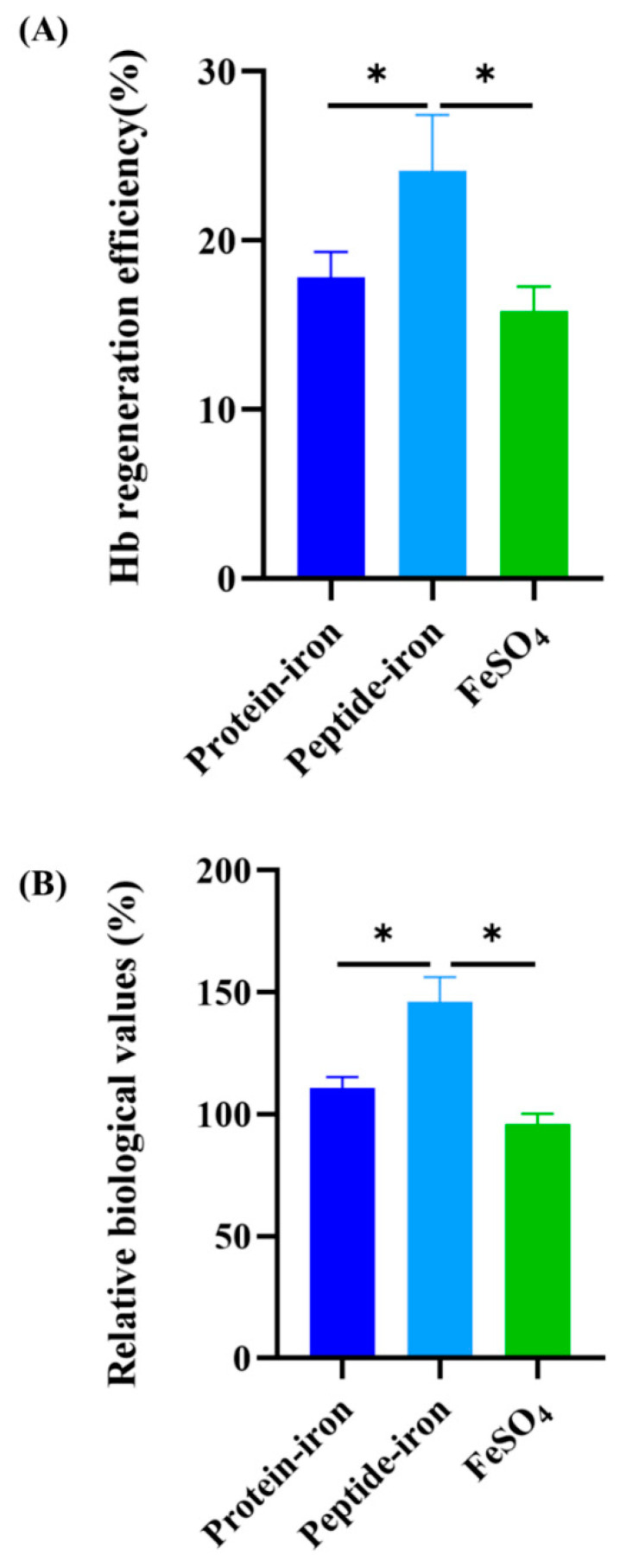
Hemoglobin regeneration efficiency and relative biological value of iron-supplement groups. (**A**) effects of different iron supplements (Antarctic krill protein–iron complex, peptide–iron complex, and FeSO_4_) on hemoglobin concentration after iron supplementation; (**B**) analysis of the relative biological value of the iron-supplemented group (Antarctic krill protein–iron complex, peptide–iron complex, and FeSO_4_ groups) using the Hb regeneration efficiency of FeSO_4_ as a reference (100%). Data are presented as mean ± SD. Significant differences are indicated by asterisks (*p* < 0.05).

Figure 4A illustrates the regeneration efficiency of Hb. Oral administration of the Antarctic krill peptide–iron complex (24.10 ± 3.33%) was more efficient than that of the protein–iron complex (17.82 ± 1.52%). The Hb regeneration efficiency of the FeSO_4_ group was lower (15.80 ± 1.46%). Hb regeneration efficiency was not significantly different between the Antarctic krill protein–iron complex group and the FeSO_4_ group (*p* > 0.05). Using the Hb regeneration efficiency of FeSO_4_ as a reference (100%), an analysis of the relative biological value of the iron-supplemented group was conducted (Figure 4B). The Antarctic krill peptide–iron complex group (152.53 ± 21.05%) had a higher relative bioavailability of iron than the protein–iron complex group (112.75 ± 9.60%) (*p* < 0.05). Of note, the Antarctic krill peptide–iron complex group had a 39.78% higher biological value than the protein–iron complex group. Combined with the data of the hematological indicators, the iron content of liver and spleen, and the hemoglobin regeneration efficiency, the Antarctic krill peptide–iron complex provides a better iron supplement compared with the protein–iron complex.

### 3.5. Expression of Iron-Regulated Genes

**Figure 5:**
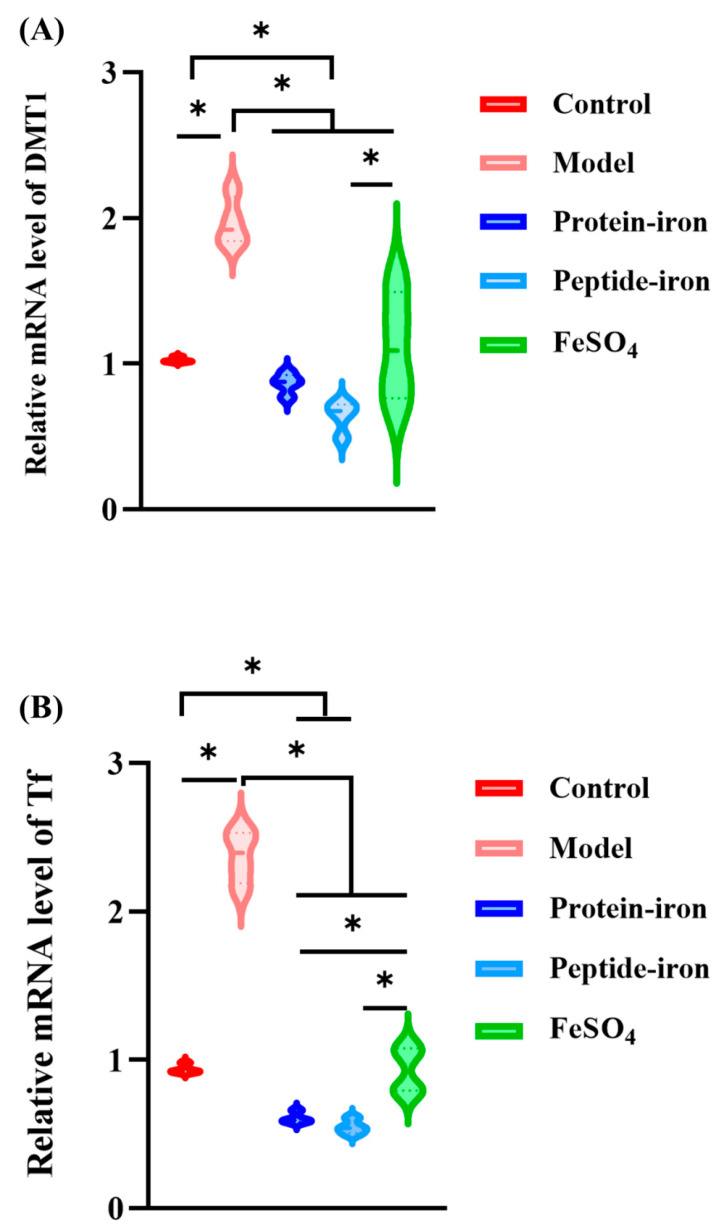

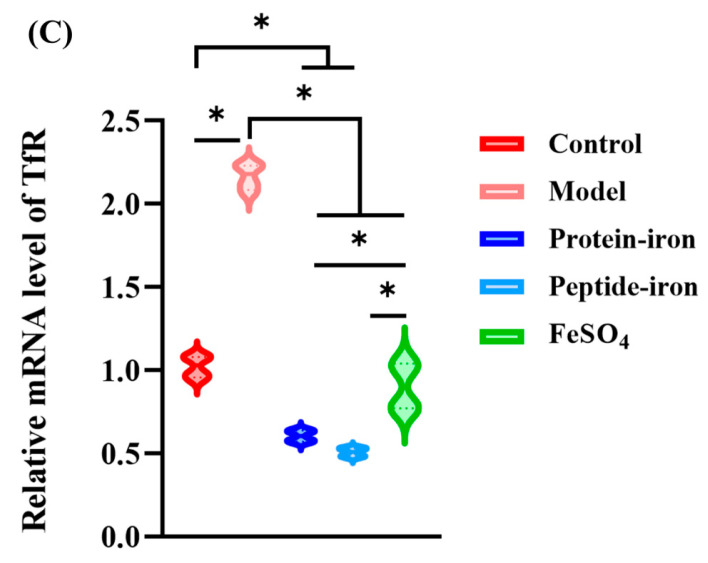
Effects of in the iron-supplemented group on expression of iron-regulated genes in the liver. (**A**) The changes of gene expression level of DMT1 of mice in the liver after iron supplementation for different groups (control, model, Antarctic krill protein–iron complex, peptide–iron complex, and FeSO_4_ groups); (**B**) the changes of gene expression level of Tf of mice in the liver after iron supplementation for different groups (control, model, Antarctic krill protein–iron complex, peptide–iron complex, and FeSO_4_ groups); (**C**) the changes of gene expression level of TfR of mice in the liver after iron supplementation for different groups (control, model, Antarctic krill protein–iron complex, peptide–iron complex, and FeSO_4_ groups). Data are presented as mean ± SD. Significant differences are indicated by asterisks (*p* < 0.05).

Non-heme iron can be transported across the apical membrane of enterocytes via the transmembrane protein DMT1 [43]. Researchers found that kidney DMT1 levels were significantly higher in rats fed iron-deficient foods and significantly lower in rats fed iron-rich foods [44]. As shown in Figure 5A, the DMT1 mRNA levels in the IDA group were significantly higher than those in the control group (*p* < 0.05). After iron supplementation, the gene expression levels of DMT1 returned to normal. The ranking of DMT1 gene expression levels was as follows: the Antarctic krill peptide–iron complex < the protein–iron complex < the FeSO_4_.

Tf originates in the liver and transports iron throughout the body to various tissues. Under iron-deficiency conditions, Tf synthesis is markedly increased via unknown mechanisms. Most cells uptake iron through Tf-bound ferric iron via TfR [45]. Tf with a high iron content binds to TfR on the surface of red blood cells and enters the cells through receptor mediated endocytosis [46,47]. As shown in Figure 5B,C, the IDA group had higher mRNA levels of Tf and TfR than the control group. The Tf and TfR gene expression levels decreased to normal in all the supplementation groups. This decrease was more rapid after administration of the Antarctic krill protein–iron complex and the peptide–iron complex as compared to that in the FeSO_4_ group, but the difference was not significant.

Regulation of DMT1, Tf, and TfR mRNA expression revealed good bioavailability of Antarctic krill protein–iron complexes, peptide–iron complexes, and FeSO_4_ in mice. At the same dose, the Antarctic krill protein–iron and peptide–iron complex groups had lower levels of DMT1, Tf, and TfR mRNA in the liver than the FeSO4 group. Furthermore, the Antarctic krill peptide–iron complex group had lower DMT1, Tf, and TfR mRNA levels than the protein–iron complex group. 

### 3.6. In Vivo Antioxidant Activities

Accumulation of radical oxygen species (ROS) can disrupt cells and their structures. In the presence of superoxide dismutase (SOD), superoxide anion was catalyzed to hydrogen peroxide (H_2_O_2_), which was then decomposed into H_2_O by catalase [48]. Additionally, glutathione peroxidase acts directly as an antioxidant by reacting with superoxide radicals, peroxyl radicals, and singlet oxygen molecules [49]. These enzymes regulate various cellular functions and protect cells against oxidative damage. Studies have reported that IDA-induced oxidative stress can damage gastric mucosal tissue [50,51]. To explore the protective effects of the Antarctic krill protein–iron and peptide–iron complexes on the gastric mucosa of IDA mice, SOD activity, glutathione peroxidase (GSH-Px) activity, and malondialdehyde (MDA) concentration in gastric tissue were assessed.

**Figure 6:**
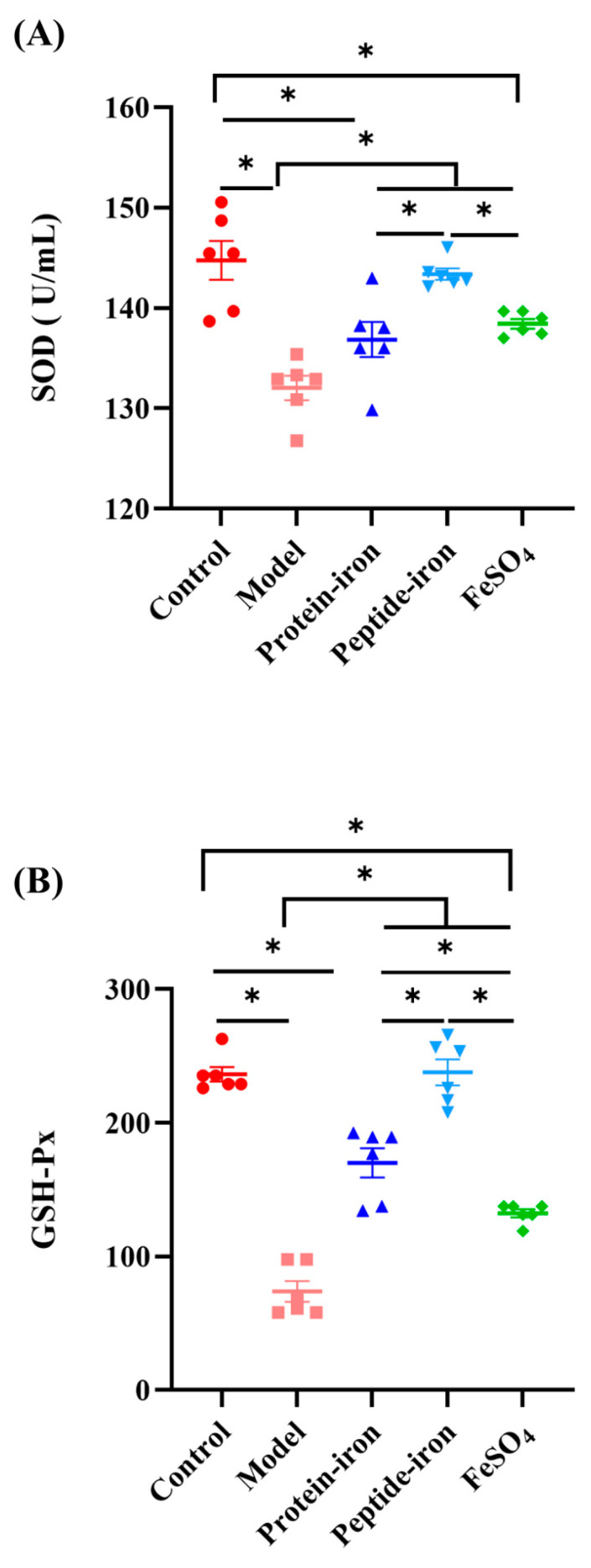

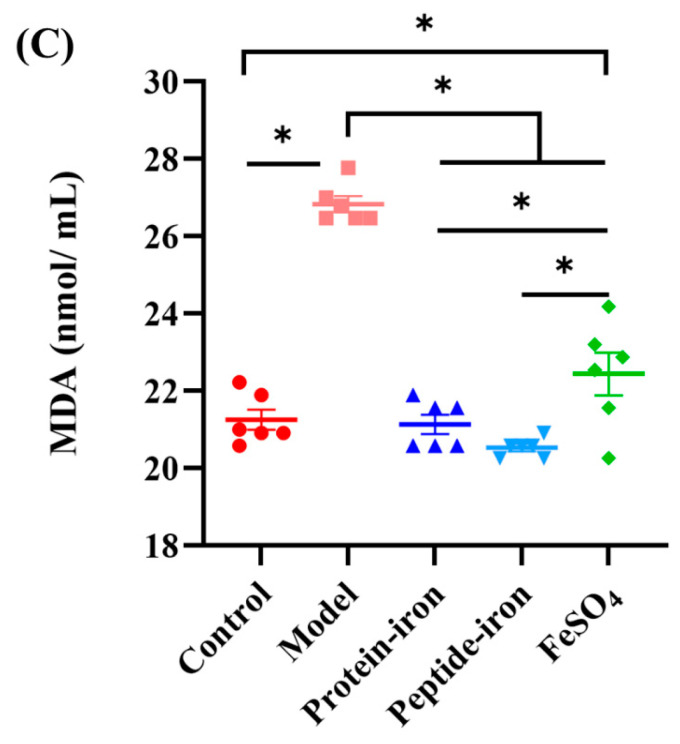
Effects of iron-supplemented group on in vivo antioxidant enzymes activity and level of mice. (**A**) The changes of SOD activity of mice in the gastric tissue after iron supplementation for different groups (control, model, Antarctic krill protein–iron complex, peptide–iron complex, and FeSO_4_ groups); (**B**) the changes of GSH-Px activity of mice in the gastric tissue after iron supplementation for different groups (control, model, Antarctic krill protein–iron complex, peptide–iron complex, and FeSO_4_ groups); (**C**) the changes of MDA concentration of mice in the gastric tissue after iron supplementation for different groups (control, model, Antarctic krill protein–iron complex, peptide–iron complex, and FeSO_4_ groups). Data are presented as mean ± SD. Significant differences are indicated by asterisks (*p* < 0.05).

The SOD activity in IDA mice (132.03 ± 2.95 U/mL) was significantly lower than that in the control (144.75 ± 4.75 U/mL) or iron-supplemented mice (Figure 6A). The SOD activity of the Antarctic krill protein–iron group (136.85 ± 4.27 U/mL) was comparable to that in the FeSO_4_ group (138.44 ± 1.17 U/mL), whereas the SOD activity of the mice in the Antarctic krill peptide–iron group (143.38 ± 1.39 U/mL) was significantly higher (*p* < 0.05). There was no significant difference between the control and Antarctic krill peptide–iron groups (*p* > 0.05). IDA mice (73.85 ± 19.07) had the lowest GSH-Px activity (*p* < 0.05) compared with normal or iron-supplemented mice (Figure 6B). In addition, the Antarctic krill peptide–iron complex group (237.84 ± 23.93) showed higher GSH-Px activity than the control group. Among the iron-supplemented mice, GSH-Px activity was lowest in the FeSO_4_ group (132. 42 ± 7.14). In general, reduced levels of SOD and GSH-Px activity are the main manifestations of IDA [52]. Our study suggests that IDA reduces the activity of antioxidant enzymes. Administration of the Antarctic krill protein–iron and peptide–iron complexes and FeSO_4_ significantly increased the activity of antioxidant enzymes, which gradually returned to near-normal levels. Compared to the FeSO_4_ treatment, the Antarctic krill protein–iron as well as peptide–iron complex treatments were more effective, and the Antarctic krill peptide–iron complex had a higher antioxidant capacity. This indicated that the antioxidant activity of the enzymatic hydrolysate obtained by proteolysis was improved. 

As a product of lipid peroxidation, MDA is typically used to estimate the extent of lipid peroxidation in biological systems [53]. Despite the extensive literature on iron and lipid peroxidation, oral iron supplementation has not been extensively studied. The control mice had an average concentration of MDA of 21.26 ± 0.64 nmol/mL (Figure 6C) which was significantly increased in IDA mice (26.83 ± 0.51 nmol/mL; *p* < 0.05). The Antarctic krill protein–iron complex group (21.13 ± 0.11 nmol/mL) and the peptide–iron complex group (20.53 ± 0.25 nmol/mL) had lower MDA concentration than the IDA group (*p* < 0.05), but a significant difference was not observed from the normal mice (*p* > 0.05). In general, mice with IDA treated with the Antarctic krill protein–iron and peptide–iron complexes showed improved antioxidant enzyme activity and reduced MDA levels, indicating that the Antarctic krill peptide–iron complex exhibits higher in vivo antioxidant activity than the protein–iron complex.

Oxidative stress can be caused by the dysregulation of iron pathways. Oxidative stress is primarily caused by the production of hydroxyl radicals and lipids [54]. Several studies have demonstrated that animal proteins and/or their antioxidant peptides may reduce oxidative stress via hydrolysis [55]. Rahimpour et al. confirmed that Antarctic krill hydrolysate has antioxidant activity [56]. Ferrous iron chelators form coordination bonds with ferrous iron, which lowers the redox potential of ferrous irons, thereby stabilizing the reduced form of ferrous ion. This property gives ferrous iron chelators an effective secondary antioxidant capacity [57]. During hydrolysis, Antarctic krill hydrolysates progressively enhance their iron-binding activity [22], and the antioxidant capacity of the Antarctic krill peptide–iron complex is higher than that of the protein–iron complex.

### 3.7. Histopathological Observation

**Figure 7:**
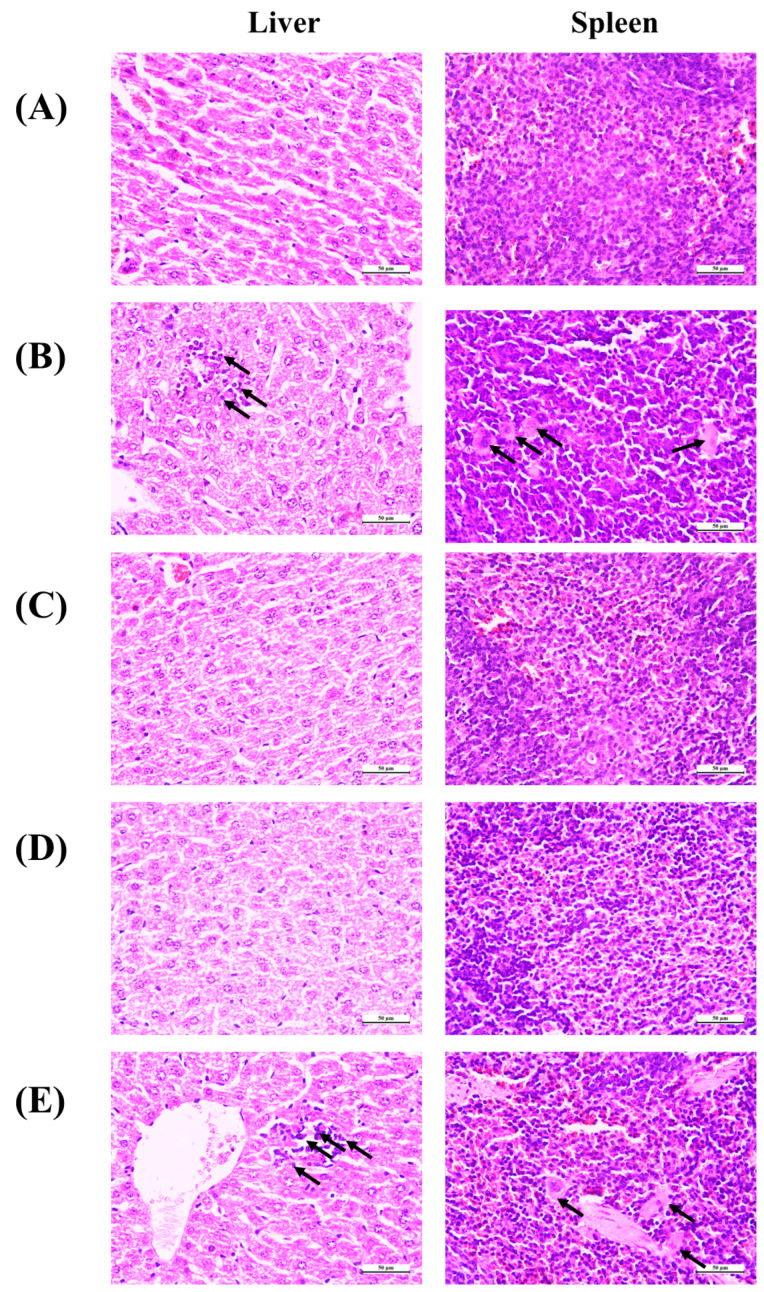
Histopathological examination of liver and spleen of mice after iron supplementation for different groups. (**A**) Representative images of liver and spleen in control group stained with H&E and photographed 40× magnifications; (**B**) representative images of liver and spleen in model group stained with H&E and photographed 40× magnifications; (**C**) representative images of liver and spleen in Antarctic krill protein–iron group stained with H&E and photographed 40× magnifications; (**D**) representative images of liver and spleen in Antarctic krill peptide–iron group stained with H&E and photographed 40× magnifications; (**E**) representative images of liver and spleen in FeSO_4_ group stained with H&E and photographed 40× magnifications. Black arrows showing monocyte infiltration in the portal area and fine hemosiderin pigment deposited in individual macrophages in the liver, and the obvious appearance of tangible body macrophages and inflammatory cells in the spleen.

To investigate the protective effect of Antarctic krill proteins and peptides on the tissue damage of liver and spleen caused by IDA, hematoxylin and eosin (H&E) staining was used in this experiment and the paraffin sections of liver and spleen were observed under a microscope. As shown in Figure 7, the histological evaluation of the liver revealed IDA-induced hepatic injury, including monocyte infiltration in the portal area and deposition of fine hemosiderin pigment in individual macrophages (black arrows). In addition, there is the phenomenon of reunion. Liver tissues from mice treated with Antarctic krill peptide–iron or protein–iron complexes showed no signs of organ damage or inflammation while the liver tissue of mice treated with FeSO_4_ showed agglomerated particles. As for the spleen tissue, the control group showed intact spleen cells under the microscope. Spleen injury caused by iron deficiency anemia is manifested by the obvious appearance of tangible body macrophages and inflammatory cells (black arrows). Moreover, the mice treated with FeSO_4_ had visible macrophages in their liver tissue. Antarctic krill protein–iron complex group and peptide–iron complex groups showed normal spleen tissue structure without detectable lesions. FeSO_4_ caused splenic lesions, confirming its toxicity.

Iron deficiency leads to oxidative stress that is closely associated with ROS [58]. Highly reactive ROS damage the cells by oxidizing and destroying biomacromolecules. Evidence suggests that diet is an important regulator of the cellular redox state and prevents oxidative stress-induced apoptosis in cells. FeSO_4_ aggravates oxidative stress in vivo and causes histopathological damage [59]. In this study, administration of the Antarctic krill peptide–iron or protein–iron complex significantly reduced the extent of iron deficiency-induced macroscopic and microscopic spleen tissue damage.

In summary, the Antarctic krill peptide–iron complex was found to be more effective than the protein–iron complex and FeSO_4_ in iron absorption and utilization by evaluating the body weight, blood parameters, iron contents in the liver and spleen of the mice. Results indicated that Antarctic krill peptide–iron complex significantly increased the hemoglobin, serum iron and iron content in the liver and spleen in iron-deficiency anemia mice (*p* < 0.05) compared with Antarctic krill protein–iron complex. Despite the gene expressions of the divalent metal transporter 1, the transferrin and transferrin receptor could be better regulated by Antarctic krill peptide–iron complex and protein–iron complex; the relative bioavailability of iron in the Antarctic krill peptide–iron complex group was significantly higher than in the protein–iron complex (*p* < 0.05). Moreover, the Antarctic krill peptide–iron complex can increase superoxide dismutase and glutathione peroxidase activity, thereby reducing malondialdehyde levels in gastric tissue compared with the protein–iron complex. These findings suggest that IDA mice benefit from iron supplementation with Antarctic krill peptide–iron complex. Furthermore, the investigation of the mechanism by which Antarctic krill peptide–iron complex improves iron deficiency is necessary.

## Figures and Tables

**Table 1:** Primers for quantitative PCR.

Gens	Primers	Sequences	Products (bp)
Tf	Forward	GCAGTGTCAGAGCACGAGAATAC	156
	Reverse	GGTCATAGCATCGGCTTCACTT	
TfR	Forward	CGTGGAGACTACTTCCGTGCTAC	139
	Reverse	GAGATACATAGGGCGACAGGAAG	
DMT1	Forward	CTGCCTACAGCAACTCATCCCT	136
	Reverse	GTGAACGCCCAGAGTTTACGA	
GAPDH	Forward	CCTCGTCCCGTAGACAAAATG	133
	Reverse	TGAGGTCAATGAAGGGGTCGT	

## Data Availability

The data supporting the results of this study are included in the present article.
